# Fibroblasts play a potential role in bone destruction *via* osteopontin related caldesmon expression and polymerization in human non-functioning pituitary adenomas

**DOI:** 10.1038/s41598-017-17679-2

**Published:** 2017-12-13

**Authors:** Li-yang Zhang, Xiao-lu Ge, Zheng Li, Yong-jian Tang, Yuan-yuan Xiong, Xue-jun Li, Jin-fang Liu, Si-yi Wanggou, Chun-tao Li, Kui Yang, Xin Chen, Zhong-Liang Hu, Yun-sheng Liu, Zhi-Xiong Liu

**Affiliations:** 1 Department of Neurosurgery, Xiangya Hospital, Central South University; 87 Xiangya Road, Changsha, Hunan 410078 P.R. China; 2 Department of Medicine, University of Oklahoma Health Science Center; 975NE, 10th ST, Oklahoma City, Oklahoma 73104 United States; 3 High Resolution Mass Spectrometry Laboratory of Advanced Research Center, Central South University, Changsha, Hunan 410008 P.R. China; 4 Department of Pathology, Xiangya Hospital, Central South University; 87 Xiangya Road, Changsha, Hunan 410078 P.R. China

## Abstract

Non-functioning pituitary adenomas (NFPAs) are the most frequent pituitary tumors. The elucidation of the mechanisms of aggressive NFPAs in bone destruction is required in order to guide the clinical diagnosis and treatment of NFPAs. In the present study, we investigated the differential proteomics of fibroblasts isolated from clinical specimens of NFPAs with or without bone destruction. Proteomic analysis revealed a group of molecules associated with cytoskeleton organization, including caldesmon, were differentially expressed between fibroblasts isolated from bone destruction NFPAs (BD-NFPAs) and fibroblasts isolated from non-bone destruction NFPAs (NBD-NFPAs). The secreted proteins analysis found that osteopontin was significantly upregulated in BD-NFPAs fibroblasts. Furthermore, immunohistochemical staining of the NFPAs clinical samples showed that the expression of caldesmon in stromal cells and the expression of osteopontin in both tumor cells and stroma were significantly increased in BD-NFPAs. Taken together, our results indicate a possible way that osteopontin secreted from both NFPA cells and surrounding fibroblasts modify caldesmon expression and polymerization in fibroblasts, which may contribute to bone destruction in NFPA patients.

## Introduction

Pituitary adenomas (PAs) are common benign monoclonal neoplasms that are associated with increased morbidity and mortality, and which account for approximately 15% of intracranial neoplasms^1,2^. The prevalence of PA is increasing in developing countries, including China^3^. Notably, some non-functioning pituitary adenomas (NFPAs) are clinically more aggressive, exemplified by bone destruction, internal carotid artery occlusion and cavernous sinus invasion. NFPA are the most frequent pituitary tumors, forming a PA subtype that is non-hormonally active^4^. NFPAs symptomatology is driven by compression, including symptoms such as optic chiasma or cavernous sinus syndrome, which are associated with various degrees of pituitary failure^5^. However, many NFPAs progress to macroadenomas due to an absence of symptoms. Unlike common NFPAs, aggressive NFPAs can be resistant to medical treatments, such as dopamine or somatostatin agonists^6^, and tend to recur and subsequently invade surrounding tissues after initial surgical resection. In the case of aggressive NFPAs, it is of clinical importance that such malignant behavior is successfully treated.

Unfortunately, the mechanism(s) underlying aggressive NFPAs still requires clarification. To date, most research has focused on the clinical management and pathogenesis of the tumor cells, including the changes in the cell genome, transcriptome and signaling transduction^7^. However, although the tumor microenvironment is considered to form ‘fertile soil’ that facilitates tumor initiation and progression^8,9^, there has been little investigation of the role of stromal cells in the NFPAs microenvironment. Fibroblasts are one of the most important stromal cells in the tumor microenvironment, and can either disperse throughout the tumor or reside in the periphery of tumors^10,11^. Fibroblasts facilitate tumor transformation and progression, including by the expression of factors involved in extracellular matrix reconstruction, as well as the promotion of angiogenesis and cell growth^12,13^. Recent work indicates that fibroblasts promote osteoclastogenesis in keratocystic odontogenic tumors, a frequent benign odontogenic tumor that occurs mainly in the jawbone, with fibroblast effects mediated *via* interaction with epithelial cells^14,15^. The current study investigates that role of fibroblasts in the bone destruction evident in aggressive NFPAs.

We previously reported that osteopontin (OPN), a bone-resorbing protein, along with caldesmon (CaD) were relevant to bone metastases in non-small cell lung cancer^16^. OPN can regulate CaD in a calcium dependent manner. The present study investigates the differential proteomics and secreted proteins of fibroblasts that were isolated from clinical NFPA cases, with (BD-NFPAs) or without (NBD-NFPAs) evidence of bone destruction. The results indicate that fibroblasts play an important role in aggressive NFPAs. OPN and CaD may clinical utility as biomarkers of aggressive NFPAs, especially BD-NFPAs.

## Results

### Biological properties of fibroblasts isolated from NBD- and BD-NFPAs

**Figure 1:**
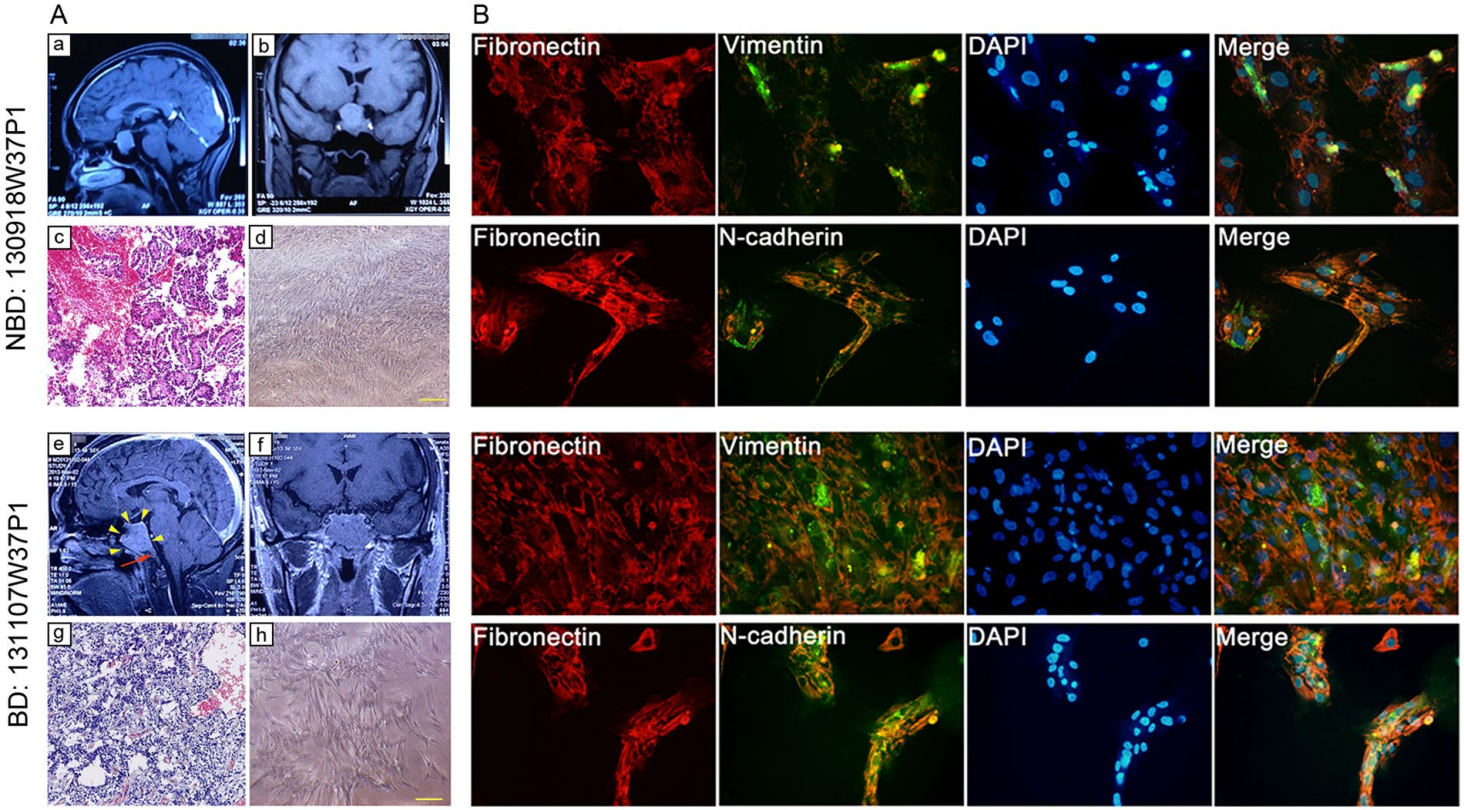
Patient-derived NBD- and BD-NFPA fibroblasts. (**A**a,**A**b,**A**e,**A**f) Brain MRI detection. (**A**c,**A**g) Patients derived tissues H&E staining. (**A**d,**A**h) Primary cultured fibroblasts of clinical cases with NBD- and BD-NFPA fibroblasts. (**B**) Immunofluorescence to detect the expression of fibronectin, vimentin, and N-cadherin in NBD- and BD-NFPA fibroblasts.

Fibroblasts were isolated from 4 BD-NFPAs and 4 NBD-NFPAs patients. MRI brains scans indicated solid mass lesions in the pituitary region. Table 1 summarizes the patients’ clinical information. For example: patient code in tissue bank: 130918W37P1, T1 scan coronal and sagittal view: solid mass lesion measuring 2.9 × 2 × 2.7 cm in saddle area, extending to the suprasellar region; compressing optic nerves; clear boundary (Fig. 1Aa,Ab); patient code in tissue bank: 131107W37P1, T1 scan coronal and sagittal view: solid mass lesion measuring 3.2 × 2.9 × 3.1 cm in saddle area, extending to suprasellar region; compressing optic nerves; irregular and ill-defined margins with surrounding tissues infiltration; dorsum selae presents obvious bone destruction (Fig. 1Ae,Af). Pituitary adenomas were identified by H&E staining for the patient-derived tissues (Fig. 1Ac,Ag). Hormonal readiness analysis proved that the tumors were non-functioning pituitary adenomas (data not shown). Fibroblasts were cultured as explants, growing as spindle-shaped, elongated fibroblast-like cells in L-DMEM (10% FBS, 1 × NEAA, without Penicillin and streptomycin) after 7 days (Fig. 1Ad,Ah). When confluence reached 90%, cells were generated and expended into a T75 flask. In appearance, there were no significant differences between the fibroblasts derived from NBD- and BD-NFPAs. To confirm that all fibroblasts were of mesenchymal origin, IF staining was utilized to show the expression of the mesenchymal cell marker, vimentin, as well as fibronectin and N-cadherin (Fig. 1B).

**Table 1:** Clinical characterization of NFPA cases for fibroblast isolation (n = 8).

Group	Tissue Bank Code	Gender	Age (yr.)	Tumor site	Diagnosis	MRI indicated
NBD-NFPA	130918W37P1	Female	41	saddle area	NFPA	Non-Bone destruction
NBD-NFPA	130930W37P1	Male	56	saddle area	NFPA	Non-Bone destruction
NBD-NFPA	140116W37P1	Male	50	saddle area and suprasellar region	NFPA	Non-Bone destruction
NBD-NFPA	140116W37P2	Male	25	saddle area	NFPA	Non-Bone destruction
BD-NFPA	131107W37P1	Male	55	saddle area and suprasellar region; invasion to sphenoid sinus	NFPA	Bone destruction of sellar floor, Saddle slope
BD-NFPA	140115W37P1	Male	57	saddle area and suprasellar region; invasion to right cavernous sinus	NFPA	Bone destruction of sellar floor
BD-NFPA	140122W35P1	Female	46	saddle area and suprasellar region; invasion to sphenoid sinus	NFPA	Bone destruction of sellar floor
BD-NFPA	140122W37P2	Male	50	saddle area and suprasellar region; invasion to sphenoid sinus	NFPA	Bone destruction of sellar floor

### Proteomic profiling identifies differential cytoskeleton organization proteins in NBD- and BD-NFPAs fibroblasts

**Figure 2:**
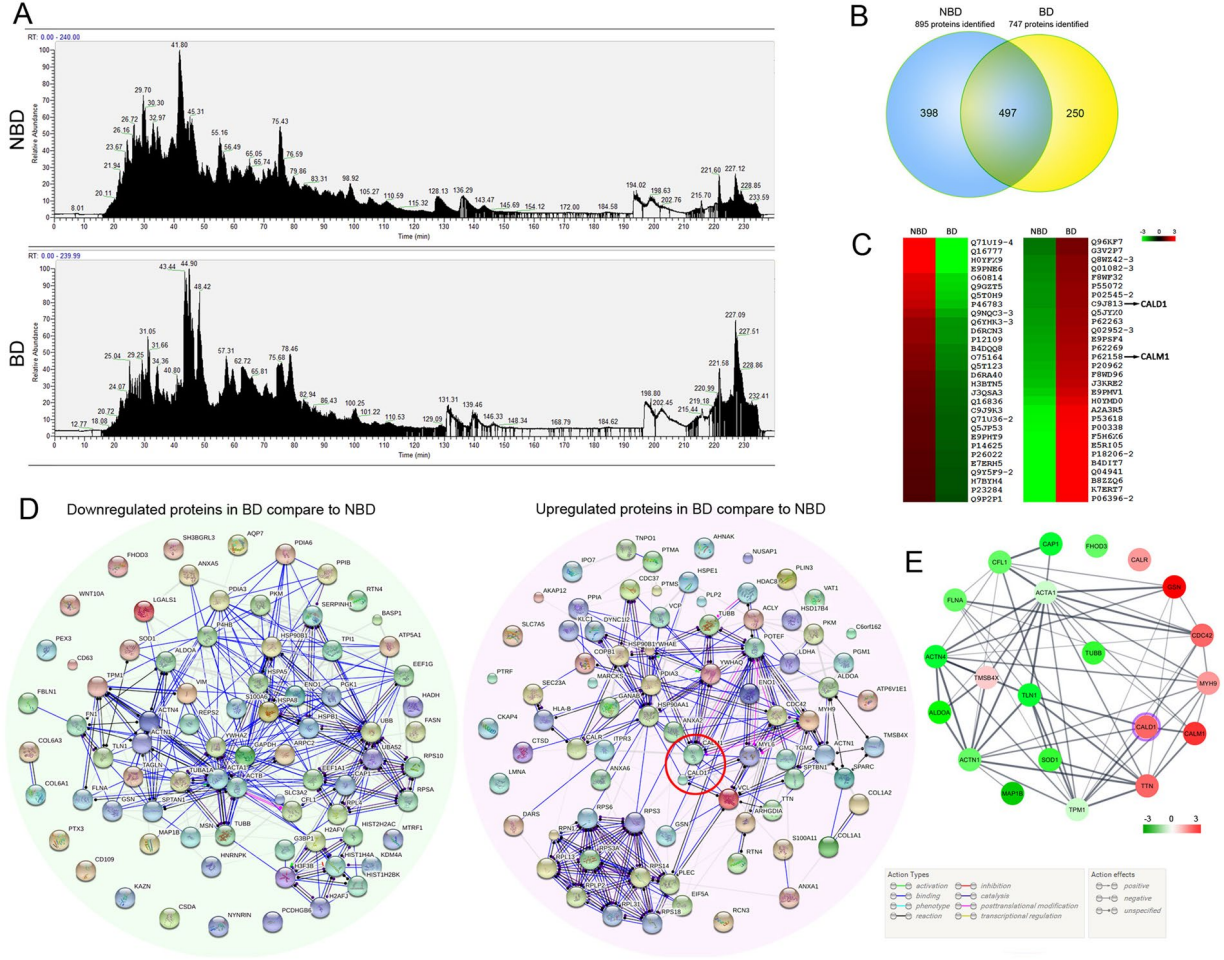
Proteomic analysis of fibroblasts isolated from NBD- and BD-NFPAs. (**A**) Total ion current (TIC) for mass chromatograms of total non-labeled proteins from the NBD-NFPA and BD-NFPA fibroblast groups. (**B**) Venn diagram compared the proteins identifications from NBD- and BD-NFPA fibroblast. (**C**) Heat-map of the relative expression of the normalized proteins in the two groups. The logged protein levels are indicated as green to red boxes, according to the color bar shown on the top right. Green indicates proteins downregulated, while red indicates proteins upregulated in BD-NFPA fibroblasts compared to NBD-NFPA fibroblasts. (**D**) Protein to protein interaction networks built by SRTING v10. The left panel shows the interaction network of downregulated proteins and the right panel shows the interaction network of upregulated proteins in BD-NFPA fibroblasts compared to NBD-NFPA fibroblasts. Red circle in the right panel indicates the central position of CALD1 and CALM1. Network edges represent molecular actions among the proteins. Color of the edge indicates action type, and line shape indicates the predicted mode of action, according to the labels shown on the bottom right. (**E**) Interaction network of differentially expressed proteins defined as cytoskeleton organization Biological process GO term built by Cytoscape 3.4.0 connected to STRING. Color of the circle indicates the logged protein levels of upregulated or downregulated in BD-NFPA fibroblasts compare to NBD-NFPA fibroblasts, according to the color bar shown on the bottom right. The purple ring indicates the position of CALD1.

To clarify the role of fibroblasts in NFPAs with or without bone destruction, HPLC-MS/MS was performed to identify the proteomics of patient derived fibroblasts. Total ion current (TIC) for mass chromatograms non-labeled proteins from NBD- and BD-NFPA fibroblasts groups (each with four samples pooled) showed significant proteomic difference between NBD- and BD-NFPA fibroblasts (Fig. 1A). A Venn diagram showed 895 and 747 proteins from NB- and BD-NFPA fibroblasts, respectively, with 497 proteins common to both groups (Fig. 1B). By employing a TMT-based quantitative proteomic approach, a significant difference between groups is indicated by a 1.2-fold change or above. Using this criterion, a group of proteins participating in cytoskeleton organization showed significant between-group differences (Fig. 2A–D). A functional annotation table, built in DAVID, showed a set of genes encoding the differentially expressed proteins in the two groups of fibroblasts, to be enriched in Biological process GO term–cytoskeleton organization, and KEGG pathway–Regulation of actin cytoskeleton (data not shown). An interaction network of proteins that participate in cytoskeleton organization processes was then built (Fig. 2E). In this network, Caldesmon (CaD), encoded by the CALD1 gene, was upregulated, with predicted associations with calmodulin (CALM1), cell division control protein 42 homolog (CDC42), alpha-actinin-1 (ACTN1), tropomyosin alpha-1 chain (TPM1) and talin-1 (TLN1).

### Increased CaD expression and polymerization in BD-NFPA fibroblasts

**Figure 3:**
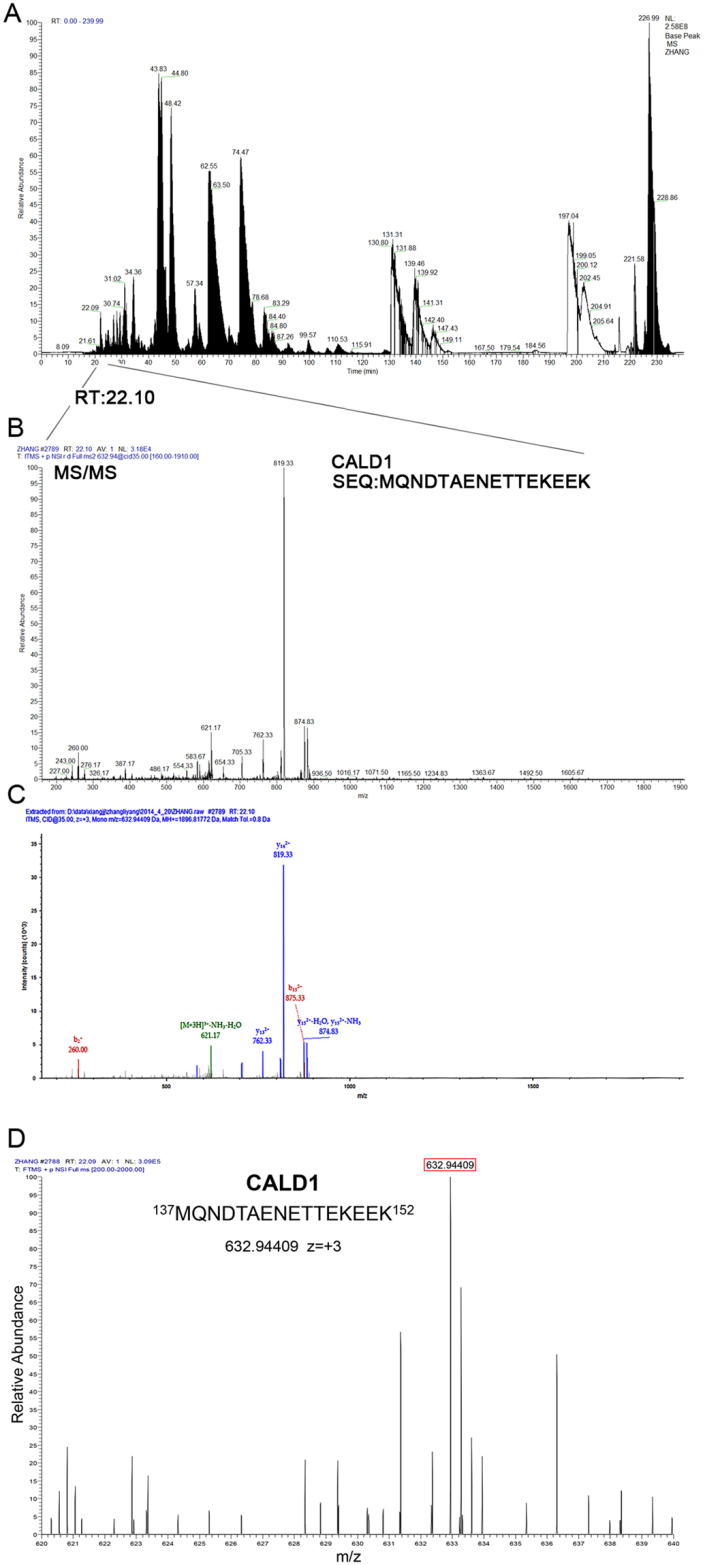
The MS/MS spectra of CALM1 and CALD1. (**A**) Base peak chromatogram from HPLC-MS/MS of a tryptic digest of primary cultured fibroblasts, of NFPA clinical cases with bone destruction, showing cell proteins group pool. (**B**) MS/MS spectrum of tryptic peptides from CALD1 and CALM1. (**C**) LC-MS/MS spectra of precursor ions m/z 632.94409 corresponding to residues 137–152 (MQNDTAENETTEKEEK) of CALD.

**Figure 4:**
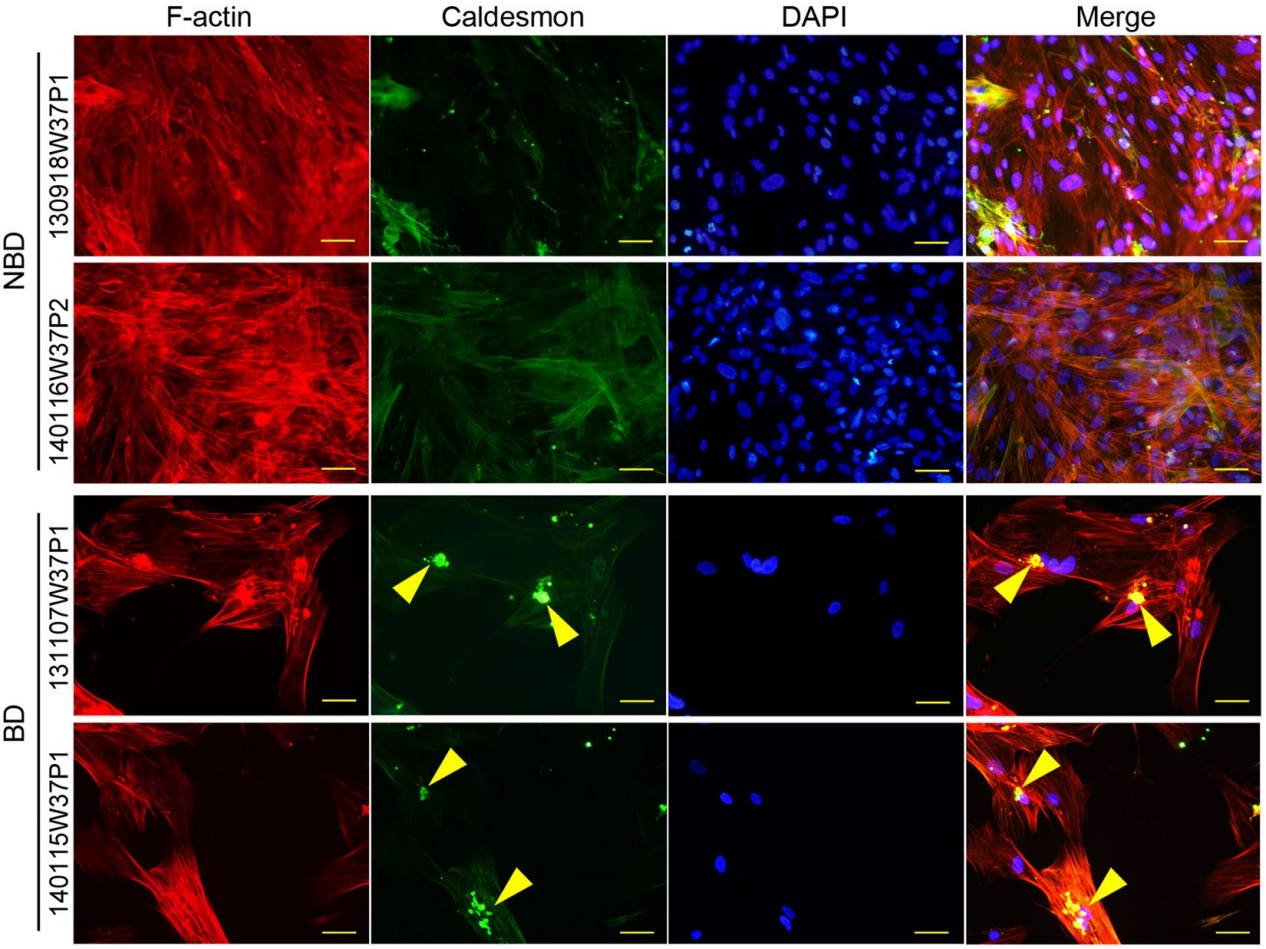
Immunofluorescence detected the expression of CALD1 in primary cultured fibroblasts of NFPA clinical cases with bone destruction and non-bone destruction. Arrow heads indicate polymerization of CaD.

As CaD was differentially upregulated in BD-NFPAs, *versus* NBD-NFPAs, fibroblasts (Fig. 3), an immunofluorescence assay was performed in order to detect CaD expression and localization in the fibroblasts of both groups. Interestingly, in the cytoplasm of BD-NFPAs fibroblasts, CaD, in colocalization with F-actin, was not only overexpressed but also polymerized. Under confocal microscopy, CaD presented as non-filamentous, compact dots in BD-NFPAs fibroblasts (Fig. 4).

### Secreted protein array analysis reveals increased OPN secretion from BD-NFPAs fibroblasts

**Figure 5:**
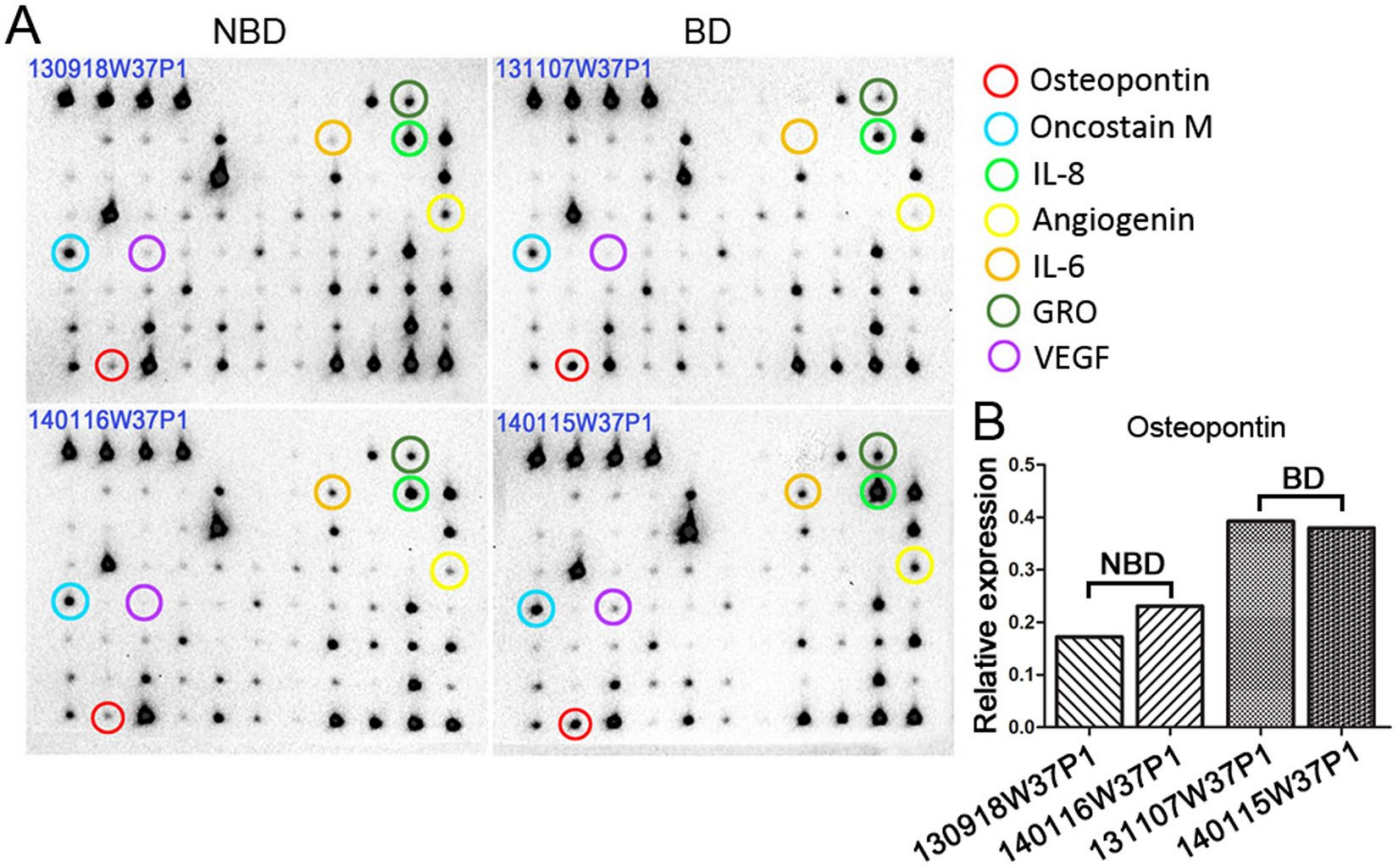
Cytokine arrays analysis of fibroblasts isolated from NBD- and BD-NFPAs. (**A**) Cytokine arrays detected secreted proteins of OPN from fibroblasts isolated from NBD- and BD-NFPAs. (**B**) Relative expression of OPN in protein arrays (n = 2, *P < 0.05).

Total protein expression profiles compared the structural and functional changes in BD-NFPAs, *versus* NBD-NFPAs, fibroblasts, including secretion profiles. To this end, the levels of 40 cytokines and chemokines in the cell culture supernatants of the NFPA fibroblasts were investigated with the RayBio Mouse Inflammation Antibody Array (RayBiotech, USA). Two cases of each group were detected. A number of cytokines showing differential secretion between groups, including OPN, oncostain M, interlukin (IL) -6, IL-8, angiogenin, GRO (C-X-C Motif Chemokine Ligand 1) and vascular endothelial growth factor (VEGF). Among these molecules, the matricellular protein, OPN, was most significantly elevated in the supernatants derived from BD-NFPA, *versus* NBD-NFPA, fibroblasts (Fig. 5).

### High CaD and OPN expression indicates bone destruction in NFPA patients

**Figure 6:**
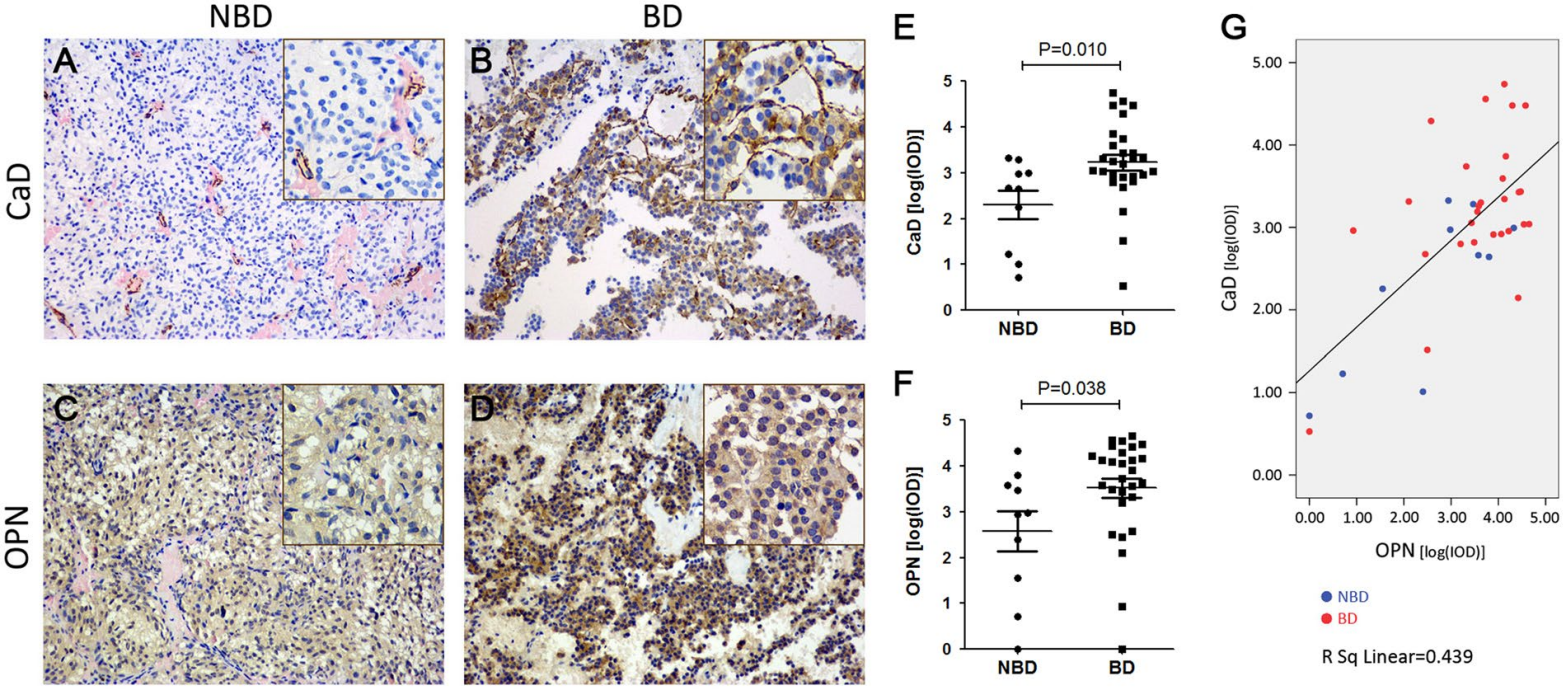
Immunohistochemical (IHC) staining of CaD and OPN in tissues specimens from NFPA patients (10 from NBD-NFPA patients and 28 from BD-NFPA patients). (**A**) IHC staining of CaD in NFPA without bone destruction, shows that CaD is expressed mainly in tumor stromal cells, especially in the vascular endothelial cells and fibroblasts. (**B**) IHC staining of CaD in NFPA with bone destruction shows much stronger staining in tumor stroma and positive staining in the cytoplasm of adenoma cells. (**C**) IHC staining of OPN in NFPA without bone destruction, shows weak positive staining in the cytoplasm of adenoma cells and tumor stroma. Much stronger staining can be being in NFPA with bone destruction (**D**). (**A**–**D**) With original magnification 200x; inner box: original magnification 400x). (**E**,**F**) Comparison of the IHC stain integrated optical density [log (IOD)] of CaD (**E**) and OPN (**F**) in NBD-NFPA and BD-NFPA specimens. (**G**) Scatter Plots shows positive correlation between IHC stain integrated optical density [log (IOD)] of CaD and OPN in NFPA specimens.

The expression of CaD and OPN in tissue specimens from NFPA patients was also by immunohistochemistry. CaD strongly stained in the tumor stroma (branch-shaped appearance), with positive staining also evident in the cytoplasm of some adenoma cells of BD-NFPA specimens. CaD staining was restricted to the fibroblasts and vascular endothelial cells, as well as some other stromal cells, in NBD-NFPA specimens. OPN was expressed both in the cytoplasm of adenoma cells and tumor stroma. OPN staining was much stronger in BD-NFPAs samples (Fig. 6A–D, Table 2). There was no relationship of CaD or OPN staining with age, gender or tumor size of the NFPA patients, but a strong correlation was evident between CaD or OPN expression levels and bone destruction (Fig. 6E,F, Table 2). In addition, a positive correlation between CaD and OPN expression was found (Pearson correlation = 0.663, P < 0.001; Fig. 6G).

**Table 2:** Correlation between CALD1 and OPN IHC scores and clinical characteristics of NFPA patients (n = 38).

Characteristics	No.	CADL1 [Log(IOD)]	P value	OPN [Log(IOD)]	P value
Gender			0.797		0.318
Male	19	3.03 ± 0.75		3.48 ± 1.11	
Female	19	2.94 ± 1.21		3.07 ± 1.37	
Age (yr)			0.412		0.607
<50	21	2.86 ± 0.87		3.18 ± 1.16	
≥50	17	3.13 ± 1.34		3.39 ± 1.38	
Tumor diameter (cm)			0.522		0.887
≤3	21	2.89 ± 1.09		3.30 ± 1.19	
>3	17	3.10 ± 0.87		3.24 ± 1.36	
Bone destruction			0.010*		0.038*
No	10	2.31 ± 0.97		2.58 ± 1.41	
Yes	28	3.23 ± 0.90		3.52 ± 1.11	

*P < 0.05.

## Discussion

Fibroblasts are cells of mesenchymal origin that produce a wide variety of matrix proteins, growth factors and proteases. Their characterization relies on morphological, proliferative and phenotypical characteristics^17^. Fibroblasts play roles in tumor stroma organization, angiogenesis and tumor cell proliferation by producing an array of factors, including extracellular matrix, angiogenic and tumor-growth-promoting factors^18^. Fibroblasts can also potentiate tumor invasion and metastasis by producing matrix-degrading enzymes, including matrix metalloproteinases, thereby aiding tumor cell dissemination. Furthermore, following appropriate inductive processes, fibroblasts can differentiate into osteoclasts, which may play a role in regulating bone reconstruction as well as homeostasis in the surrounding tissue^14^. Arthritic synovial fibroblasts can induce osteoclast formation causing bone destruction^19^.

For the first time in the present study, fibroblasts from NFPAs were isolated, with fibroblast protein expression patterns compared in those with, *versus* without, bone destruction. Findings indicate that BD-NFPAs fibroblasts produce high-levels of OPN, which is a glycoprotein generally produced by osteoclasts. This phenomenon might indicate a osteoclast-mimic capability of BD-NFPAs fibroblasts. Meanwhile, the total proteins derived from BD-NFPAs, versus NBD-NFPAs, fibroblasts indicate a different pattern of cytoskeleton organization processes. The current study focused on the cytoskeletal regulatory protein CaD, which was reported to have correlated expression with OPN in non-small cell lung cancer (NSCLC) biopsies in our previous study. Both OPN and CALD1 had higher expression levels in specimens of NSCLC patients, in comparison to non-cancerous control specimens, with their expression levels being even higher in patients showing evidence of metastasis^16^. CaD is a multimodular protein encoded by the CALD1 gene, which regulates contractility and actin cytoskeleton remodeling in smooth muscle and nonmuscle cells^20^. CaD exists as two isoforms, a high molecular mass caldesmon (h-CaD) that is expressed in smooth muscle, and a low molecular mass caldesmon (l-CaD), which is more ubiquitously distributed, including in dedifferentiated SMCs^21,22^. The serum l-CaD level is considered to be a good discriminator between glioma patients *versus* patients with other intracranial tumors, as well as discriminating other neurologic diseases in comparison to healthy controls^23^. The differential expression of splicing variants of CALD1 is closely related to modulation of the glioma vasculature^21,24^.

In the last 2 decades, OPN has been proposed to serve as a biomarker of tumor progression and metastasis, including in breast, lung, gastric, colon, hepatic and prostate carcinomas^16,25–29^, with the circulating osteopontin reported to be a dual marker of bone destruction and angiogenesis in multiple myeloma^30^. OPN serves both a cell attachment function and a cell signaling function^31^. It can influence intracellular calcium levels in different types of cells *via* its interaction with the αvβ3 integrin. Zimolo *et al*.^32^ observed a transient increase in intracellular calcium levels in both rat osteoclast and mouse-derived osteoclast-like cells exposed to OPN. Tanabe *et al*.^33^ found that OPN increased the proportion of osteoclasts exhibiting transient elevations in cytosolic Ca^2+^ (oscillations). The same phenomenon was observed in nasopharyngeal carcinoma cells when treated with OPN, although mixed results in different cell types are evident^31,34^. Importantly, the intracellular Ca2 + concentration increase can upregulate the expression of CaD and enhance CaD polymerization^16^. Previous data also shows that stromal cells in the tumor microenvironment can translocate CaD to podosomes in a Ca2 + /calmodulin manner and promote the metastatic ability of nasopharyngeal carcinoma (NPC) cells through invadopodia formation, with which the NPC cells degrade the extracellular matrix^35^.

Overall, the current study shows that CaD and OPN were relatively highly expressed in the NFPAs associated with bone destruction. CaD, effectively restricted in tumor stromal cells, showed higher expressed and polymerization properties in BD-NFPA fibroblasts. Likewise, OPN had an elevated expression level in tumor stroma as well as in adenoma cells of BD-NFPAs cases, with CaD and OPN levels showing a positive correlation. Given that CaD is regulated by the intracellular Ca^2+^ concentration, OPN may be modulating CaD expression and polymerization pattern via Ca^2+^ regulation.

## Material and Methods

### Human Non-functional pituitary adenoma tissue collection

We employed 38 non-functional pituitary adenomas patients’ surgical tissues from Human pituitary adenoma tissue bank of Department of Neurosurgery, Xiangya Hospital (XYNS): 10 cases without bone destruction and 28 cases with bone destruction. Pathological diagnosis and clinical information, e.g: gender, age, sex, tumor site and imagological diagnosis, were obtained from both of medical records department and XYNS tissue bank of Xiangya Hospital. Written informed consent was acquired from all of the patients that were enrolled in this paper. All experimental protocols were approved by the ethical review committee of Xiangya Hospital. All methods were carried out in accordance with relevant guidelines and regulations.

### Cell culture

Fibroblasts derived from human pituitary adenoma tissues were obtained from patients who underwent surgery in the Department of Neurosurgery, Xiangya Hospital of Central South University. The isolation and culture of the NFPA fibroblasts were performed using the previously described methods^36^. NFPA fibroblasts were grown in L-DMEM medium supplemented with penicillin G (100 U/mL), streptomycin (100 mg/mL), 1% NEAA (100 × NEAA, Hyclone, USA.), 1% sodium pyruvate (100 × sodium pyruvate, Hyclone, USA.) 10% fetal calf serum at 37 °C with 5% CO_2_.

### Harvesting of secreted proteins of NFPA fibroblasts from conditioned medium and total proteins from NFPA fibroblasts

Cells were grown to approximately 80% confluence (approximately 3 × 106 cells) in 150-mm culture dishes (Corning Inc., Corning, NY, USA), washed three times, with pre-heated at 37 °C DPBS, then washed two times with pre-heated at 37 °C 10 ml serum-free L-DMEM medium, and incubated in serum-free medium at 37 °C for 48 h. After incubation, the conditioned mediums were collected and centrifuged at 1700 × g for 15 min to eliminate suspended cells. Then the supernatants were centrifuged at 10,000 × g for 30 min without brake down. After high-speed centrifuge, the supernatants were concentrated and desalted using Amicon Ultra-15 tubes (molecular mass cutoff, 3000 Da; Millipore, Billerica, MA, USA), followed by addition of a proteinase inhibitor cocktail (1 mM phenylmethylsulfonyl fluoride [PMSF], 1 mM benzamidine, 0.5 μg/ml leupeptin). Protein concentrations of supernatants were determined using the BCA protein assay reagent (Thermo Scientific Pierce Rockford, IL, USA). The collected conditioned media were then stored a −80 °C until use.

After harvesting the conditioned mediums, cells were collected in mRIPA buffer containing protease inhibitors (50 mM Tris, pH 7.4; 100 mM NaCl; 1% Nonidet P-40; 0.5% deoxycholic acid; 0.1% SDS; 10 μg/ml of aprotinin; 10 μg/ml of leupeptin and 1 mM PMSF). Subsequently, cells were placed on ice for 30 min without ultra-sonic, then High-speed centrifuged at 10,000 × g, with supernatants removed into new 1.5 ml EP tubes. 2D-clear-up kit (GE Healthcare, USA.) was employed to desalt, degrease and enrich proteins.

### TMT-coupled high-performance liquid chromatography (HPLC)-MS/MS analysis

#### Protein digestion and TMT labeling

Different mixed samples from the same group (4 samples of NBD-NFPA fibroblasts group and 4 samples of BD-NFPA fibroblasts group) at a ratio of 1:1:1:1, individually, and measured for concentrated low-abundance proteins with ProteoMiner protein enrichment kit (Bio-Rad, California, USA). Total protein was extracted using a protein extraction buffer consisting of 7 M urea, 2 M Thiourea, 4% Chaps, 1% DTT, and 0.5% (v/v) protease inhibitor cocktail. According to the manufacturer’s instructions of TMT Isobaric Mass Tag Labeling kit (Thermo, USA), protein pellets (100 μg of each sample) were resuspended in 100 mM triethylammonium bicarbonate (TEAB) with 200 mM Tris (2-carboxyethyl) phosphine hydrochloride (TCEP) and incubated for 1 h at 55 °C. After addition of 5 μL of 375 mM iodoacetamide (IAA), the samples were incubated for 30 min at room temperature in the dark, then digested overnight with trypsin at 37 °C (Promega). Each 100 µg sample was labeled with 41 µl of the TMT Label Reagents following the manufacturer’s protocol. The NBD-NFPA fibroblasts sample was labeled with reporter tag 126, whilst the BD-NFPA fibroblasts sample was labeled with reporter tag 127. Following labeling, the peptide mixtures were pooled and desalinated for LC-MS/MS analysis.

#### LC-Mass Spectrometry

Labeled peptides were analyzed by nano-flow liquid chromatography (Nano-LC)/electrospray ionization (ESI)-tandem MS (MS/MS) using the UltiMateTM 3000 RSLCnano system online coupled to a linear trap quadrupole (LTQ)-Orbitrap Velos Pro mass spectrometer (Thermo Fisher Scientific, Massachusetts, USA). Peptide mixtures were dissolved in 0.1% formic acid. Separation of peptides was carried out as follows: peptide mixtures were loaded onto one C18 pre-columns (30 µm × 100 mm, Thermo Fisher Scientific, Massachusetts, USA) equilibrated with 0.1% (v/v) trifuoroacetic acid, washed and pre-concentrated for 5 min at a flow rate of 0.3 µL/min. The pre-column was then switched in line with a C18 RP nano LC column (150 mm × 75 μm, 2 μm, 100 Å, Thermo Fisher Scientific, Massachusetts, USA) and peptides were eluted with a binary system consisting of solvent A (0.1% formic acid in aqueous phase) and solvent B (0.08% formic acid in 80% ACN) with a flow rate of 0.3 μL/min. The elution linear gradient was as follow: (a) 3% B in 0–5 min, (b) 3–40% B in 5–70 min, (c) 95% B in 75–80 min, (d) 3% B in 81–90 min. The LTQ-Orbitrap Velos Pro instrument was externally calibrated using LTQ Velos ESI Positive Ion Calibration Solution (Thermo Fisher Scientific, Massachusetts, USA). The general mass spectrometric parameters were as follows: spray voltage, 2.2 kV; capillary voltage, 4.5 V; capillary temperature, 250 °C; tube lens voltage, 100 V. For data-dependent MS/MS analyses, the software Xcalibur (Thermo Fisher Scientific, Massachusetts, USA) was used. Full scan MS spectra were acquired at a mass resolution of 60,000 (mass range 100–2000 m/z) in the Orbitrap analyzer.

#### Data Processing

Proteome Discoverer 1.4 software (Thermo Scientifc, Waltham, MA, USA) and UniProt KB/Swiss-Prot database (release 2014_10) performed to analyze and search the MS data. The error window for precursor and fragment ion mass values was set to 10 ppm and 0.8 Da, respectively. The number of allowed missed cleavage sites for trypsin was set to two, and phosphorylation (STY), oxidation (M), deamidation (NQ), and carbamidomethylation (C) were all selected as variable modifications. TMT-labeled peptide amino terminus and TMT-labeled lysine (+229.163 Da) were also set as variable modifications. The false discovery rate (FDR) for peptide was set to 1% by applying the target-decoy strategy. A common contaminants database was also included for quality control. Proteins that met the following criteria were considered differentially expressed proteins: (i) proteins were identified based on ≥ 2 peptides with ≥ 95% confidence and (ii) proteins were considered decreased when the protein levels demonstrated an averaged ratio-fold change ≤ 0.8 in the LC-MS/MS analyses. Unsupervised hierarchical clustering of normalized and mean-centered was performed by using Gene Cluster 3.0 with an average linkage clustering method and viewed by TreeView version 1.60. The protein–protein network was built in STRING (http://string-db.org, version 10)^37^, and experimental predictions of high confidence (0.700) were transferred to Cytoscape v3.4.0 for network visualization^38^. Gene ontology (GO) enrichment for biological processes and KEGG pathway was performed on the human proteome using the Database for Annotation, Visualization and Integrated Discovery (DAVID Bioinformatics Resources 6.8; https://david.ncifcrf.gov/home.jsp).

#### Immunofluorescence

The cells were fixed in pre-warmed 37 °C 4%PFA for 15 min, then washed cell twice with PBS, blocked and permeabilised in 5% bovine serum albumin (BSA)/0.1% Triton × 100 in antibody buffer (150 mM NaCl, 50 mM Tris base, 2% BSA, 100 mM L-lysine and 0.04% Na azide, pH 7.4) for 60 min at room temperature. Primary antibodies directed against L-caldesmon, fibronectin, vimentin, or N-cadherin were diluted in antibody buffer (1:1000). The cells were incubated in primary antibody solution overnight at 4 °C, followed by applying fluorescently-conjugated secondary antibody, then rinsed with PBS three times and then mounted in a mounting medium containing DAPI. F-actin was stained with Aexa594-phalloidin. The cells were viewed using a fluorescence microscope (Olympus, Japan).

#### Immunohistochemistry

Paraffin-embedded blocks of NFPA patient tissues were sliced into 4-μm-thick sections for immunohistochemical (IHC) staining using antibodies against caldesmon (1:300 dilution; Abcam) or OPN (1:150 dilution; Origene) antibodies. IHC analyses were performed using an automatic IHC staining system according to the manufacturer’s instructions (Bond, Vision BioSystems). The intensity of IHC staining was detected and analyzed using Image Pro Plus version 6.0 software (Media Cybernetics, USA). At least three isolated sights with magnification of 200x were picked randomly from each slide for integrated optical density (IOD) detection. Log-transformed mean IOD value of each slide was used for further statistical analysis.

### Statistical analysis

Numerical data are presented as mean ± standard deviation (SD). SPSS software (version 13.0; Chicago, IL, USA) was used for statistical analysis. Independent sample t-test was used to compare data between two groups. Pearson correlation was used to analyze the correlation of expression of caldesmon and OPN. All statistical tests were two-sided. Differences were considered statistically significant at P < 0.05.
